# Advanced Methods for Detection of *Bacillus cereus* and Its Pathogenic Factors

**DOI:** 10.3390/s20092667

**Published:** 2020-05-07

**Authors:** Nalini Ramarao, Seav-Ly Tran, Marco Marin, Jasmina Vidic

**Affiliations:** INRAE, AgroParisTech, Micalis Institute, Université Paris-Saclay, 78350 Jouy-en-Josas, France; seav-ly.tran@inrae.fr (S.-L.T.); marco.marin@inrae.fr (M.M.)

**Keywords:** *Bacillus cereus*, bacterial detection, biosensors, toxin detection, food safety, human health

## Abstract

*Bacillus cereus* is an opportunistic foodborne pathogen causing food intoxication and infectious diseases. Different toxins and pathogenic factors are responsible for diarrheal syndrome, like nonhemolytic enterotoxin Nhe, hemolytic enterotoxin Hbl, enterotoxin FM and cytotoxin K, while emetic syndrome is caused by the depsipeptide cereulide toxin. The traditional method of *B. cereus* detection is based on the bacterial culturing onto selective agars and cells enumeration. In addition, molecular and chemical methods are proposed for toxin gene profiling, toxin quantification and strain screening for defined virulence factors. Finally, some advanced biosensors such as phage-based, cell-based, immunosensors and DNA biosensors have been elaborated to enable affordable, sensitive, user-friendly and rapid detection of specific *B. cereus* strains. This review intends to both illustrate the state of the *B. cereus* diagnostic field and to highlight additional research that is still at the development level.

## 1. Introduction

Since the beginning of the 20th century, the role of *Bacillus cereus* in food-poisoning has been clearly demonstrated [1,2,3]. In Europe, it is the second cause (as confirmed and suspected causative agent) of the foodborne outbreak (FBO) after *Staphylococcus aureus* [4]. In 2017, 44% of the reported cases of FBO in France were caused by *B. cereus*. Nevertheless, the impact of *B. cereus* in FBO is most likely underestimated due to the lack of systematic surveillance and is often misdiagnosed as *S. aureus* or *Clostridium perfringens* symptom-like infections.

*B. cereus* can cause emetic or diarrheal-type of foodborne illnesses, which are generally mild and self-limiting. However, severe systemic infections have also been associated with this pathogen. Although rare, these infections may lead to the death of newborns and immunologically-compromised or vulnerable individuals [4,5].

The pathogenicity potential of *B. cereus* is extremely variable, with strains being harmless and others lethal. This variability makes it difficult to communicate around the dangerousness of *B. cereus* and often leads to misunderstanding and mismanagement of the associated risks. Therefore, the characterization of *B. cereus* potential of pathogenicity is a major challenge for the agri–food industries and hospitals. Due to the lack of validated and standardized analytical methods to assess the presence of specific toxins, only the presence of presumptive *B. cereus* is usually examined. Therefore, during the last decade, the elaboration of new diagnostic tools for toxin gene profiling and for toxin quantification has gained increasing importance. It is thought that the application of such new methods will lead to a significant improvement of *B. cereus* diagnostics and patient care. In this review, we provide an overview of the traditional methods currently used in the food industry and at hospitals to detect *B. cereus*. We also present advanced methods developed at a bench-scale that allow a specific, rapid and sensitive detection of *B. cereus* strains and their pathogenic factors. We recently reviewed methods for the detection of *B. cereus* spores [6].

## 2. Pathogenicity of *Bacillus cereus*

### 2.1. Bacillus cereus Group

**Figure 1:**
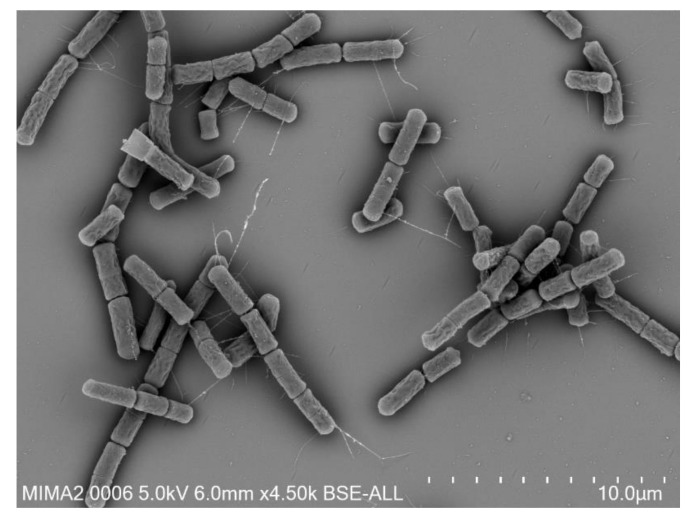
Scanning electron microscopy image of *B. cereus* cells.

*B. cereus* are spore-forming, Gram positive, aerobic or facultative anaerobic bacteria. The presence of a peritrichous ciliature allows the bacteria to be motile [7]. Morphologically, *B. cereus* strains are thin, straight or slightly curved bacilli of 1 × 3–4 µm with square ends, which can form chains (Figure 1).

*B. cereus sensus stricto* belongs to a larger group of bacteria commonly named the *Bacillus cereus* group. The group is constituted of eight species: *B. mycoides*, *B. pseudomycoides*, *B. weihenstephanensis*, *B. anthracis*, *B. thuringiensis*, *B. cereus sensu stricto* (usually called *B. cereus*), *B. cytotoxicus* and *B. toyonensis*. Recently, the group was extended with new species, which were all isolated from marine sediments: *B. paranthracis*, *B. pacificus*, *B. tropicus*, *B. albus*, *B. mobilis*, *B. luti*, *B. proteolyticus*, *B. nitratireducens* and *B. paramycoides* [8], while some other strains are under evaluation [6]. Differentiation of species within the *B. cereus* group is complex due to the genetic proximity between the members of the group [9,10]. Originally, species in the group were classified on the basis of phenotypic differences, distinct virulence trait and the presence of extrachromosomal elements that reflect the specie’s virulence spectrum. Currently, the most widespread classification system of this bacteria group is based on the sequencing of *panC* housekeeping gene, which encodes for the pantoate-beta-alanine ligase C [11]. Using this classification, seven phylogenetic groups have been determined based principally on their range of growth temperatures. *B. cereus* can be found in several clusters, among which the groups III and VII comprise of species associated with high cytotoxicity [12].

*B. cereus* are ubiquitous bacteria. Besides the soil which is their primary reservoir, they can be isolated from vegetation and waters [13,14] and can colonize insects and mammals [15,16,17]. From the environment, they can be transferred into various raw materials used in the food industry. The host is contaminated by spores or vegetative cells present in ingested food, inhaled air, or entering the body through a wound. In addition, these microorganisms can deteriorate the organoleptic qualities of food (in particular eggs and pasteurized milk) with an impact on the market quality of the products, which must then be destroyed. *B. cereus*, therefore, constitutes a real public health problem and represents a major economic risk for the food industry.

However, currently, no legislation requires systematic screening of food items for this pathogen contamination. Within the EU, the only regulation implying a safety limit for *B. cereus* in foods concern dried infant formulae with an established maximum limit of 50 CFU/g (Commission regulation (EC) No 1441/2007). In France, the regulatory limit of *B. cereus* presence in food, especially starch-rich foods have been set to 1 × 10^5^ CFU/g of food (Act DGAL/MUS/N2009-8188, 07 July 2009). According to the Codex Alimentarius Commission of the Food and Agriculture Organization of the United Nations (FAO) and the World Health Organization (WHO) Codex standard for infant formula, the maximum acceptable number of *B. cereus* is 10^2^ CFU/mL [18]. Most cases of FBO due to *B. cereus* have been associated with a bacterial concentration above 10^5^ CFU/g of food material but some cases have been linked with bacterial concentration as low as 10^3^ CFU/g [2,19]. Moreover, the determination of a safety limit is difficult, as the pathogenicity does not depend solely on the number of bacterial cells [20,21,22,23].

### 2.2. B. cereus Gastro-Intestinal Infections

As previously mentioned, there are two types of food poisoning due to *B. cereus*, which differ in terms of symptoms and origin, known as the emetic and the diarrheal syndromes. The latter is associated with the production of enterotoxins in the small intestine following the ingestion of contaminated food. Bacteria can be ingested as vegetative cells or as spores, which once in the intestine, germinate, multiply, and produce enterotoxins [6,16,24]. Symptoms appear 5 h to 24 h after ingestion, and manifest through abdominal pain and profuse diarrhea [14]. Manifestations are generally mild with spontaneous remission within 24 h without any treatment. However, fatal cases resulting for example from necrotic enteritis have been reported [25].

The emetic syndrome results from an intoxication provoked by the emetic toxin cereulide, a toxin often pre-formed in food before ingestion [26]. This toxin causes nausea and vomiting that occur quickly (between 30 min and 6 h after ingestion) and has been linked to severe clinical forms associated with fatalities, in particular, because of liver failure [27,28,29,30].

### 2.3. B. cereus Non Gastro-Intestinal Infections

In addition to FBO, *B. cereus* is responsible for a variety of local and systemic infections, which can lead to the death of patients in approximately 10% of cases [5,7]. These clinical infections have undeniable repercussions on public health, in particular for fragile populations such as premature newborns. In addition, several cases of fulminant infections with *B. cereus*, similar to anthrax, and affecting healthy persons, have also been reported [31].

However, our recent epidemiological study, together with the alert launched by the Paris hospitals (AP-HP) and the health institutions have shown that the number of cases of serious *B. cereus* infections is largely underestimated and underdocumented, while the first-line treatment is not always suitable [5]. Many hospitals face deaths of newborns from *B. cereus*-related bacteremia or meningitis [32,33,34].

Taken together, *B. cereus* toxigenic bacteria are on the rise and have to be considered as emerging threats, especially for high-risk population, where adequate methods for their detection remain scarce.

## 3. Detection of *B. cereus*

### 3.1. Traditional Methods

The traditional method for *B. cereus* detection is agar plate-based counting for which the guidelines are provided by the ISO 7932:2004 that had been last revised nearly 15 years ago. Several laborious sub-points are needed, like: sample homogenization, numerous centrifugations and serial dilution preparations, all carried out in aseptic conditions to avoid any contamination [35]. The plating steps have to be carried out in duplicate for each dilution using specific media. Then, plates need to be incubated at 30 °C for 24 h to 48 h. Finally, colonies have to be streaked onto the Brain Heart Infusion (BHI) agar medium. Moreover, an additional confirmation assay is required in order to differentiate bacteria of the *B. cereus* group. This last step takes another 2 h to 24 h. Traditional methods are, thus, time-consuming, laborious, expensive and require trained staff capable of ensuring the correct procedure. Furthermore, with these methodologies, it is not possible to detect injured or viable but not-culturable (VBNC) cells. The ISO 7932:2004 method specifies a horizontal test for the enumeration of presumptive *B. cereus* cells. This method consists of a serial dilution of the samples and a subsequent dilution spreading on *B. cereus* selective Mossel medium agar (MYP). Incubation of the colonies at 30 °C during 18–24 h is needed. On MYP medium, colonies of *B. cereus* have a pink–purple color, surrounded by a characteristic halo formed of pink precipitation, which permits their identification. Finally, a hemolysis test is performed on bacterial colonies to confirm for *B. cereus* strains. This method confirms only presumptive *B. cereus* cells because not all strains of *B. cereus* are hemolytic.

More recently, alternative methods based on the NF EN ISO 7932 standard for the enumeration of presumptive *B. cereus* in food have been validated. The AFNOR BKR 23-06-02-10 and AFNOR AES-10/10-07/10 methods use a selective chromogenic medium, COMPASS and BACARA respectively. After hydrolyzation of the chromogenic substrate, *B. cereus* colonies appear in green (COMPASS) or in orange surrounded by an opaque halo (BACARA). These selective agars inhibit the majority of the background microflora and allow easy identification. Their specificity, selectivity and accuracy are comparable to the reference method. In addition, they are time-saving and do not involve a confirmation step.

### 3.2. Molecular Methods

Molecular methods used for *B. cereus* identification provide many advantages compared to the traditional methods such as versatility, lower time and resource consumption, and high specificity [36]. In the past decades, different techniques based on polymerase chain reaction (PCR) have been developed for *B. cereus* detection, such as Nested PCR [37], Randomly Amplified Polymorphic DNA PCR (RAPD PCR) [38] and Real-Time PCR (RTi-PCR) [39]. The amplification step is coupled to different electrophoresis procedures such as Pulse Field Gel Electrophoresis (PFGE) [40], Temporal Temperature Gradient gel Electrophoresis (TTGE) [41] and Denaturing Gradient Gel Electrophoresis (DGGE) [42]. The coliphage M13 sequence-based polymerase chain reaction (M13-PCR) and DNA amplification fingerprinting were applied to obtain profiles of various *B. cereus* strains [43]. The resulting discriminative and informative profiles have permitted us to trace the spread of *B. cereus* contamination in a food plant. Hall et al., [38] were able to characterize the microbiological hazard of products provided by vending machines with the use of PCR and RAPD PCR. Using specific primers that target the *gyrB* gene encoding DNA gyrase subunit B, they found out that almost 90% of bacteria isolated from hot chocolate powder and hot chocolate drinks of vending machine belonged to *B. cereus* group. In another study, Fernandez-No et al., [39] were able to simultaneously quantify *B. cereus*, *B. subtilis* and *B. licheniformis* by means of RTi-PCR. By using probes targeting the 16S rRNA gene of each bacterium, the RTi-PCR could detect each specific strains with a limit of detection (LOD) of 165 CFU/g of an artificially contaminated pasteurized foodstuff.

Over the years, new methods have been developed with the leading principle to detect and distinguish *B. cereus* from other Bacillus group members by a time-saving and in-situ analysis. For example, Manzano et al., [41] compared different molecular methods that use specific probes and primers as recognition elements to distinguish *B. cereus* from *B. thuringiensis* from different origins (food, clinical and bio-pesticide). They used three different strategies: a PCR-TTGE technique targeting the *gyrB* gene, a rep-PCR in which two specific DNA sequences were targeted, and a RAPD-PCR uses M13 primers. They demonstrated that only the rep-PCR was able to cluster strains of the same origin [41]. This work highlights the difficulty to differentiate *B. cereus* and *B. thuringiensis* even with molecular methods. Noguera and Ibarra [44] designed universal primers to amplify *cry* genes. As *B. thuringiensis* is the only strain within the *B. cereus* group to produce the cry toxin proteins, this technique has permitted to discriminate *B. thuringiensis* from other *B. cereus* strains [44]. Another method used to distinguish *B. cereus* from other members of the *B. cereus* group is the genotyping using high-resolution melting analysis [45]. This analysis allows the discrimination of *B. cereus* by typing an amplified polymorphic 16S-23S intergenic spacer region. This method has been proposed for the control of food safety following adequate sanitizing treatments. Nevertheless, the method cannot dissociate *B. cereus* from *B. thuringiensis*.

**Figure 2:**
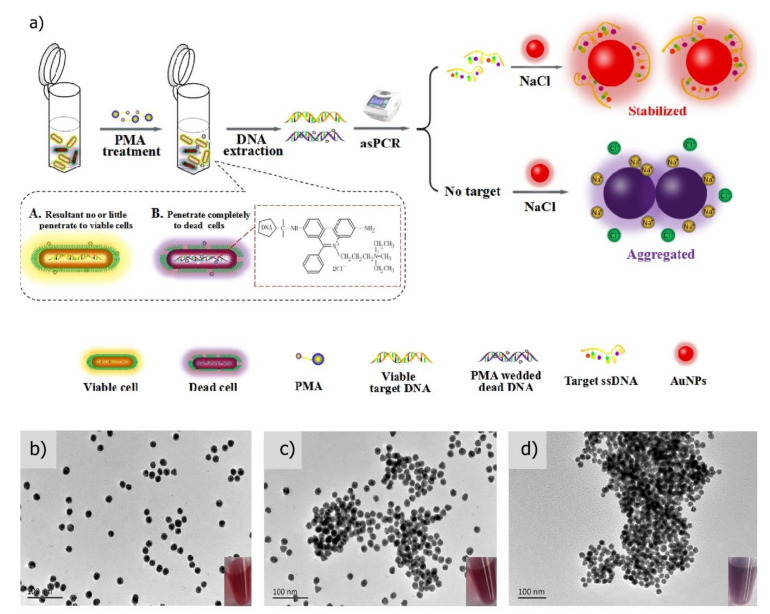
Schematic illustration of the colorimetric method based on PMA-asPCR (**a**) and visualization of gold nanoparticles for the detection of viable emetic *B. cereus*. Transmission electron microscope images correspond to (**b**) AuNPs alone, (**c**) AuNPs treated with 1 pmol ssDNA and 25 mM NaCl, and (**d**) AuNPs upon the addition of 25 mM NaCl without ssDNA. Insets show the respective images. (Reprinted with Permission from [36]).

Recently, an innovative approach to increase the rapidity of post-PCR step has been proposed by Li et al., [36]. They replaced the detection of PCR products using the laborious electrophoretic method by a rapid visualization system using gold nanoparticles (AuNPs). They also applied propidium monoazide (PMA) prior to performing the asymmetric PCR (asPCR) amplification in order to only detect the viable *B. cereus* cells. PMA is a viability-photoreactive DNA binding dye that can penetrate inside the cell when the membrane permeability is altered. Thus, it can only bind to the DNA of dead cells. PMA, upon binding, permanently modifies DNA which in consequence cannot be amplified during the asPCR step. The cereulide synthetase encoding gene (*cesB*) was used as a specific *B. cereus* biomarker targeted during the asPCR. The PCR products were mixed with AuNPs and the resulting coloration used as an indicator of *B. cereus* presence (Figure 2). Indeed, upon salt addition and in the absence of amplicons, AuNPs aggregates and form a violet precipitate. In contrast, salt addition does not induce AuNP aggregation in the presence of the amplicons and the solution remained red. Using this protocol, a LOD of 9.2 × 10^1^ CFU/mL in PBS and 3.4 × 10^2^ CFU/mL in milk were respectively obtained. Besides demonstrating the feasibility of the test, this study pointed out the crucial parameters necessary to obtain a low LOD such as the salt concentration that induces aggregation of AuNPs and the length of the primers.

Despite the advantages offered by the molecular techniques, to overtake the traditional method drawbacks, such as rapid bacterial identification at lower analysis cost, the former exhibits several limitations. Indeed, trained professionals are required to perform this type of assays, which are still laborious (PCR test takes from 2 h to 24 h) and costly. Sometimes amplification errors occur, or false-negative results may be obtained when using complex matrices such as blood or food samples [46,47,48]. Another limitation of PCR is the use of laborious, time-consuming and low-resolution gel electrophoresis methods to visualize PCR products. One exception is microfluidic PCR chips that automatize protocol by combining sample processing, DNA extraction, enzymatic amplification and detection [49,50]. Qiu et al., [51] described an inexpensive PCR module constituted of a thermal cycler combined with a PCR chip that allows amplification and real-time fluorescence monitoring which can be used for the detection of *B. cereus* genomic DNA. In addition, primers can be conjugated with biotin and dioxigenin to enable lateral flow-based detection of amplicons as an alternative to the optical measurement. Using this module, the negative sample exhibited neither a fluorescent signal nor band. They also optimized the module to allow for the pre-storage of paraffin-encapsulated PCR reagents inside the PCR chamber to achieve the device automation. Thus, reagents can be released during the heating after the melting of paraffin and initiate amplification. This lab-on-a-chip PCR module carried all the necessary steps from sample-introduction to result-visualization.

Nevertheless, molecular methods are often fall short of providing an instant, point-of-care detection, and thus, they are not suitable for safety testing in agri-food chains. Especially, it is difficult to integrate PCR based methods into a point-of-care format because they require a thermal cycler instrument to amplify the target sequence. To overcome this technical obstacle, nucleic acid sequence amplification through isothermal methods have been developed with the use of enzymes for strand separation instead of repeated heating [52,53]. Xia et al., [54] developed a loop-mediated isothermal amplification (LAMP) method for simultaneous amplification of *B. cereus* and four other foodborne bacterial pathogens *Escherichia coli*, *Salmonella enterica*, *Vibrio fluvialis* and *V. parahaemolyticus*. In their device, reagents and samples were loaded into the reaction chambers, through inlets and microchannels, to reach the Peltier heater that maintains a temperature of 64 °C during the LAMP reactions. A portable CCD camera is used to capture the fluorescence of formed amplicants. Under optimized conditions, the simultaneous detection of all five pathogens was performed in 60 min and with a success rate of 100%.

### 3.3. Biosensors

In the last few years, biosensors have emerged as ideal methods for the detection of foodborne pathogens [6,52,53,55,56,57,58,59]. The success of these miniaturized devices is mainly due to their rapidity to provide results, simplicity, and possibility of point-of-care diagnosis together with the low cost and high reliability of analysis [46,60]. In addition, only a small amount of sample is required to perform the analysis [61,62]. So far, several types of biosensors have been used for the detection of *B. cereus.* Particularly, DNA-based biosensors have had great success, as they give the possibility for selective identification of different *B. cereus* strains [63].

**Figure 3:**
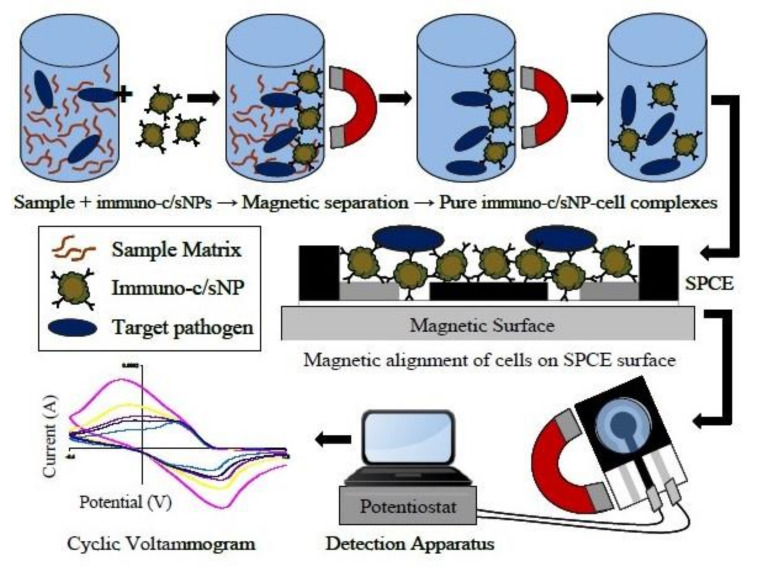
Immunomagnetic separation from sample matrix, magnetic alignment on SPCE surface and electrochemical detection of *B. cereus* cells (from [65]).

Mono or poly-clonal antibodies targeting *B. cereus* cells can also be used as recognition elements in biosensors as an alternative to DNA probes [64]. Different strategies have been carried out for *B. cereus* detection using specific antibodies. For instance, Setterington and Alocilja [65] developed a biosensor that associates the immune-magnetic separation of bacterial cells with a cyclic voltammetry detection method (Figure 3). First, magnetic/polyaniline core/shell nanoparticles decorated with a polyclonal anti-*B. cereus* antibody were incubated with the sample containing *B. cereus*. Second, a magnetic field separation was applied to collect the bacterial cells bound to magnetic particles and separate them from the background matrix (Figure 3). Finally, the collected magnetic particles were transferred to screen-printed carbon electrodes for voltammetric detection. An LOD as low as 40 CFU/mL was obtained with a pure bacterial culture. Unfortunately, this technique showed a limit in the efficiency of the bacterial collection during the immune-magnetic separation step. In response, the authors recommended that any unknown sample should be both diluted and concentrated by several orders of magnitude before the detection step to prevent false-negative signals.

The disadvantage of antibody-based detection is that commercial anti-*B. cereus* antibodies are costly and have relatively low affinity. Moreover, their stability and binding capacity can be affected by pH or temperature variations. Bacteriophages and bacteriophage-derived proteins become attractive alternatives to antibodies because of their high host specificity, high binding affinity and marked resistance to chemicals, a variation of pH and temperature. Bacteriophages and their proteins (receptors) can be successfully applied as recognition elements in biosensors for bacterial detection [66]. A *B. cereus*-specific bacteriophage endolysin cell wall-binding domain (CBD) has been used to develop a highly specific and sensitive surface plasmon resonance biosensor for the detection of *B. cereus* [67]. This biosensor has permitted us to detect *B. cereus* at concentrations of 10^5^–10^8^ CFU/ml, and showed an LOD of 10^2^ CFU/ml in pure culture. The low LOD was obtained by coupling a subtractive inhibition assay to the biosensor. For the inhibition assay, bacterial cells were pre-incubated with bacteriophage cell wall-binding domain, thus, only non-bound cells were detected by the sensor, which overcomes the limited sensitivity of the technique due to the direct capture of the bacteria. Using this strategy, a LOD of 10^3^ CFU/ml of *B. cereus* was obtained for the detection of the bacteria in contaminated cooked rice. Park et al., [68] also highlighted the superior affinity of bacteriophage CBD for *B. cereus* cells compared to commercial antibodies. Based on this idea, they developed an assay that combined the efficient and specific CBD-conjugated magnetic bacterial separation and the ATP bioluminescence bacterial detection. Using this association, they obtained a LOD as low as 10^1^ CFU/mL. In a recent study, Kong et al., [69] combined CBD and antibody to develop a lateral flow test for the cost-effective and efficient detection of *B. cereus*. The sensitivity of the strip was 10^4^ CFU/ml and the overall assay time was 20 min. Thanks to the high specificity of CBD toward *B. cereus*, the test showed no significant cross-reactivity.

Recent works employing different detection strategies and recognition elements to detect *B. cereus* cells are given in Table 1.

## 4. Detection of *B. cereus* Toxins

### 4.1. Detection of Cereulide and the Emetic B. cereus Strains

**Figure 4:**
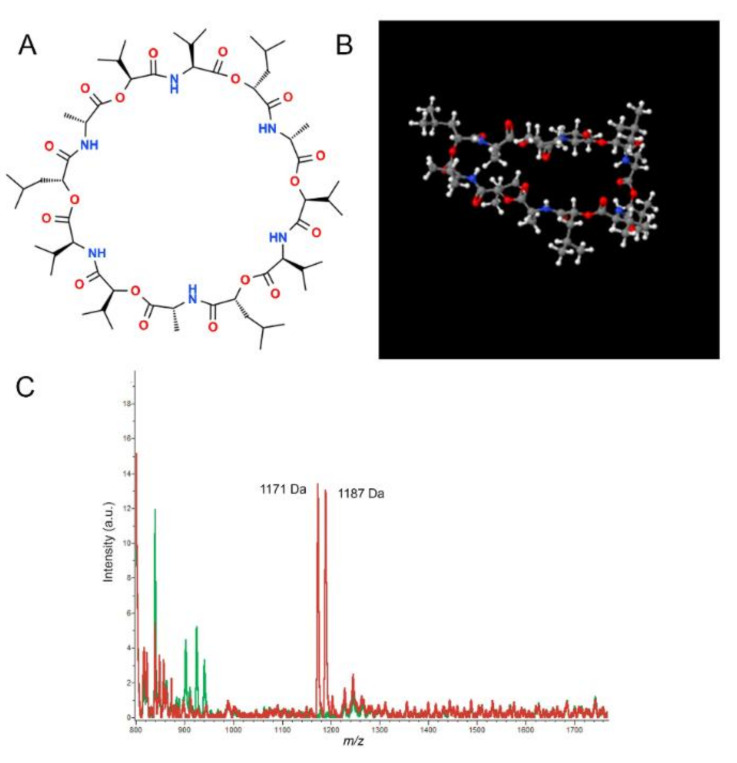
The 2D (**A**) and 3D (**B**) chemical structure of cereulide from ChemSpider (Royal Chemical Society). (**C**) Mass spectra of *B. cereus* strains/isolates, emetic strain in red, non-emetic strains in green (from [86]).

Emetic *B. cereus* strains induce the emetic food intoxication form through the production of a cereulide toxin [73]. The toxin is synthesized by a non-ribosomal cereulide synthetase enzyme encoded by the *ces* gene [22]. The toxic molecule is a cyclic depsipeptide composed of three repeats of “D-Oxy-Leu-Dala-L-Oxy-Val-L-Val” amino acids sequence (Figure 4A,B). Recently, 18 cereulide variants were reported and differences in toxicity amongst them were highlighted [22].

The efficiency of cereulide production varies between emetic strains with the distinction of no/low, medium and high producers producing respectively <1–50 ng, >50–500 ng and >500–1600 ng/mg of bacterial mass [22,74,75]. According to studies carried out on animals, poisonings appeared after ingestion-doses between 8–10 µg/kg of body weight [75,76]. In a recent FBO, concentrations of cereulide ranging from 2 to 6 µg/g of food provoked profuse-vomiting among high-risk population groups [77]. Furthermore, Decleer et al., [78] have demonstrated that subemetic doses of the toxin are sufficient to alter the mitochondrial activity and affect health. However, strains carrying the *ces* gene represent a minority, generally 1% or less, of isolates from food or the environment.

Cereulide is highly stable and resistant to treatments used by the food industry (pH, enzyme or high-temperature) [79]. In the past, its quantification in foodstuffs was hampered by the absence of suitable standard methods. Antibody-based strategies were explored but, due to the lack of cereulide immunogen properties and the low reproducibility of such tests, they are not commonly used. The cytotoxicity-based methods measure toxin-induced cell damage. Different types of cells were utilized as cereulide target, such as HEp-2 (Human carcinoma of the larynx) [80], CHO (Chinese Hamster Ovary) cells [81] or rat liver cells [82]. A distinctive bioassay based on boar sperm developed by Anderson et al., [83] aimed to detect emetic *B. cereus* through the sperm motility inhibition caused by the cereulide exposure. It allowed a qualitative detection of emetic *B. cereus* directly from the primary culture plates. To evaluate the feasibility in real conditions, the test was evaluated with bacteria isolated from rice, vegetarian dishes, cakes and pasta. The detection limit was found to be 0.3 +/− 1 ng per assay. However, this semi-quantitative bioassay could lack specificity because food matrices may contain molecules with lytic properties.

Chemical methods, like LC-MS (liquid chromatography–mass spectrometry) and Matrix-Assisted Laser Desorption Ionization–Time of Flight (MALDI-TOF), provide low cross-reactivity and high specificity for the quantification of cereulide. These measurements need a chemical standard to quantify the molecule [79]. Commercially available valinomycin was used because of its chemical structure similarity to cereulide. However, quantification of the toxin expressed as valinomycin equivalent has been a contentious point because of the differences between the two molecules in biological and chemical assays. To overcome this drawback, Biesta-Peter et al., [84], tested a synthetic cereulide as an alternative to valinomycin. They showed a faster inhibition of the boar semen mobility using the synthetic cereulide compared to valinomycin. They also demonstrated a similar cytotoxic effect against HEp-2 cells of the natural and synthetic cereulide. This new internal standard associated with LC-MS, was further tested for the quantitative detection of cereulide in food. This methodology has enabled the detection of cereulide toxin in spiked food matrices (cooked rice, chinese noodle and french fries) with a LOD of 4.1 µg/kg of sample. The feasibility of LC-MS/MS method to investigate the presence of cereulide in food leftovers and human samples associated with an outbreak was tested and reported by Delbrassinne et al., [77]. In their report, they combined plate agar counting, PCR strategies and LC-MS/MS to detect emetic *B. cereus* strain and/or its toxin. A similar methodology was previously used to evaluate the safety of dishes collected in various restaurants [85]. The detected levels of cereulide causing the outbreak were between 3.1 and 4.2 µL/g of foodstuff. Alone, LC-MS/MS, quantitatively detected the toxin in the analyzed samples with a LOD of 1 ng/g which indicates that the method was well adapted for food screening.

Zuberovic Muratovic et al., [79] applied UPLC-ESI-MS/MS (ultra-high-performance liquid chromatography–electrospray-tandem mass spectrometry) in a validation study for quantitative cereulide detection in rice and pasta matrices, using ^13^C_6_-cereulide as an internal standard together with the synthetic cereulide standard. The use of natural cereulide as standard eliminates the possible difference that may be generated by the synthetic toxin. The method provided a high specificity and sensitivity as it allows a LOD of 0.1 ng/g.

Recently, MALDI-TOF mass spectroscopy was shown to provide an easy and sensitive approach to differentiate emetic and non-emetic *B. cereus* (Figure 4C) and to quantify cereulide [86]. To develop the methodology, Ducrest et al., [87] screened for emetic toxin and potential other biomarker peaks until the mass to charge ration (*m/z*) of 20,000. They detected two characteristic peaks related to sodium and potassium alkali adducts of cereulide at 1175 and 1191 respectively. Thus, these findings accentuated the importance of the buffer used for the extent of the alkali adduct recorded since the prevalence of one rather than the other varied in various buffers. An additional peak at *m/z* 1205 was identified as cereulide variant. Finally, they demonstrated that using a Laser Desorption/Ionization–Time of Flight (LDI-TOF) instead of MALDI-TOF mass spectrometry, it was possible to ameliorate the LOD from 30 ng/mL to 30 pg/mL in milk-rice and ready-to-eat meals. Overall, chemical methods are demonstrated to detect and identify rapidly and with a high sensibility emetic *B. cereus*.

The exploitation of chemical methods enabled the establishment of a recent EN-ISO 18465 standard method based on stable isotope mass spectrometry (MS)-based dilution and synthetic cereulide as an intern standard. Although the method allows accurate quantification of cereulide in food, it shows many limitations such as being expensive, reliant on sophisticated instrumentations and experienced persons to performed and interpreted the analyzes.

**Figure 5:**
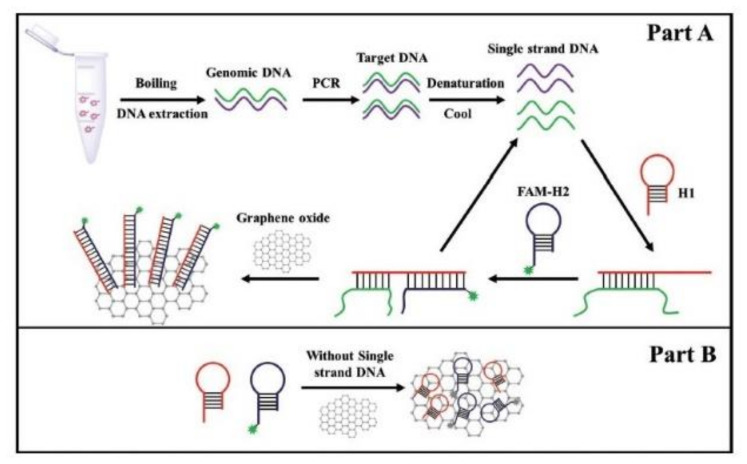
The principle of catalytic hairpin assembly-graphene oxide assay to detect emetic *B. cereus* in the presence of the target ssDNA (**A**) and in the negative control (**B**), (Adapted with permission from [88]).

Molecular methods have also been applied to detect the presence of genes from the *ces* locus that is responsible for the cereulide synthesis. For instance, Yabutani et al., [73] built two sets of primers as recognition elements in order to specifically detect emetic *B. cereus* in rice products. Applying RTi-PCR, they were able to obtain a LOD of 10^4^ CFU/g. Yu et al., [89] developed a rapid, sensitive, and specific fluorescent molecular method for emetic *B. cereus*. The method combined hybridization chain reaction (HCR), an isothermal nucleic acid amplification system, and magnetic beads-based flow cytometric bead assay (MB-based FCBA). The use of specific primers and two fluorescent hairpin probes provided high accuracy in discriminating emetic strains. A target ssDNA generated by asymmetric PCR (aPCR) was isolated onto streptavidin-MBs using a biotin-labeled primer that specifically recognized the target DNA. Then, this system was used to initiate a cascade of hybridization processes between the two fluorescent hairpin probes that allowed the formation of a double-stranded DNA polymer carrying numerous fluorophores. The amplified signal produced by this method was visualized by flow cytometry which had the advantage of measuring the fluorescence intensity of a single MB. This method has an LOD of 7.6 and 9.2 × 10^2^ CFU/mL of emetic *B. cereus* in pure culture and spiked milk, respectively. The same team modified this detection strategy to microplate format in order to simplify the read-out of the reaction [88]. To detect emetic *B. cereus* cells in milk, they combined PCR, catalytic hairpin assembly (CHA) and fluorescence-quenching property of graphene oxide (GO) (Figure 5). CHA is an enzyme-free amplification technique that achieves amplification by hybridization and strand-exchange reactions. In CHA an initiator strand triggers the formation of a duplex by a hairpin pair, then the initiator strands is released and is free to trigger subsequent CHA reaction. In the study, a classical PCR was first used to amplify the *ces* fragment. Then, the target ssDNA products were isolated and mixed with a pair of hairpin probes (H1 and H2). H1 is specific to the *ces* biomarker therefore they can hybridize together. Binding of the H1 probe to the ssDNA products opened up the probe hairpin structure, which allows the binding of H2 onto it. Hybridization of H1 andH2 induced the release of the ssDNA from the complex. The free ssDNA can then be recycled and used for another ssDNA-H1 reaction. Consequently, multiple H1-H2 duplexes were generated. Because the dsDNA had a low affinity with the GO surface, the H1-H2 duplexes do not interact with the GO and a strong fluorescence can be easily recorded. On the contrary, in the absence of target ssDNA, the two hairpin probes did not hybridize together and therefore strongly bound to the surface of the GO, which caused quenching of the fluorescence. Emetic *B. cereus* cells were detected in spiked milk samples with a LOD of 6.2 × 10^1^ CFU/mL and 5.9 × 10^2^ CFU/mL in pure culture and in spiked milk, respectively. This assay can be adapted for point-of-care detection.

A summary of the methods used for the detection of cereulide is displayed in Table 2.

### 4.2. Detection of Diarrheal Toxins

In contrast to the highly stable emetic toxin, enterotoxins produced by *B. cereus* are destroyed during food processing or when the food is reheated because they are heat-labile and susceptible to protease activity. Because of their instability, enterotoxins are usually detected in foods by molecular methods that evidence their genes [92,94,95]. Alternatively, *B. cereus* strains have to be cultivated and enterotoxins are identified in culture supernatants.

Three enterotoxins have been associated with the diarrheal syndrome, the non-hemolytic enterotoxin (Nhe), the hemolysin BL (Hbl) and the Cytotoxin K (CytK1, CytK2). Nhe complex is composed of three different proteins, A, B and C, that are encoded by the *nheA*, *nheB* and *nheC* genes, respectively [21]. Nhe is recognized as the major diarrheal toxin of *B. cereus* because of the strong correlation between cytotoxicity and concentration of Nhe in culture filtrates collected from a large collection of isolates [96]. Additionally, Guinebretière et al., showed that strains with a high production of Nhe are more often associated with food implicated in FBO than with food not associated with any illness [97]. Hbl is composed of a binding protein B encoded by the *hblA* gene and two lytic components L1 and L2 encoded by *hblC* and *hblD* genes, respectively. Hbl was the first enterotoxin identified in *B. cereus* [98]. Hbl is haemolytic, dermonecrotic and cytotoxic towards enterocytes and various other human cells [99,100,101,102,103,104]. CytK is a β-barrel pore-forming toxin. There are two variants of CytK that share 89% of identity: CytK-1, and CytK2. CytK1 was identified in 1998 from a strain of *B. cereus* that caused a large FBO in France and that conducted to the death of three elderly people [25]. Although CytK2 is five times less toxic than CytK-1, it seems more frequently associated with strains causing FBO [105].

Several PCR techniques such as mRTi-PCR [91], duplex-PCR [106], PMA-mPCR [88] and PCR coupled with electric chip [107] were used for the detection of one or multiple enterotoxin-encoding genes in food matrices [90,95,108]. Several other authors applied PCR based-strategy to detect enterotoxin-encoded genes together with the *ces* gene [90,92,109]. These methods used one or several high-affinity set of primers that specifically bind to a selected sequence present inside the gene that encodes for the toxin selected as biomarkers of diarrheal strains. So far in all collections tested, all strains carry at least one component of the *nhe* operon, while, *cytK-1* gene prevalence is low and is only present in strains of the species *B. cytotoxicus* and cytK-2 gene is carried by 42% of the strains causing FBO [110].

**Figure 6:**
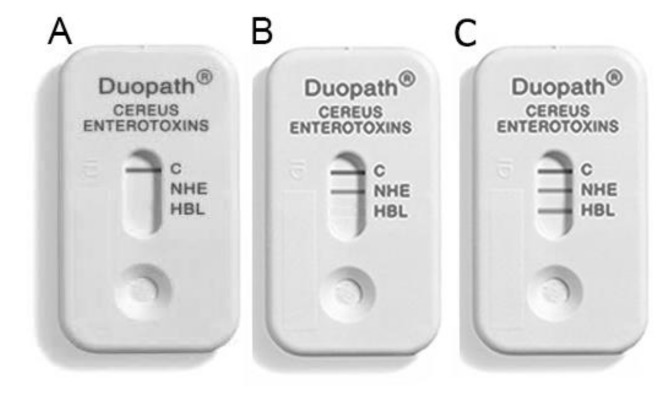
Example of Duopath^®^ lateral flow kit that detected the absence of enterotoxin (**A**), the presence of Nhe (**B**) and the presence of both Nhe and Hbl enterotoxins (**C**).

Three immunoassay kits can detect Nhe and Hbl complexes in the culture supernatant of *B. cereus*: Bacillus diarrheal enterotoxin visual immunoassay (BDE VIATM) kit (3M Tecra), *B. cereus* enterotoxin reversed passive latex agglutination (BCET-RPLA) kit (Oxoid), and the Duopath^®^ Cereus Enterotoxins (Merck). These three kits are based on lateral flow immunological detection, which allows a fast point-of-need analysis. Figure 6 illustrates the Duopath^®^ Cereus Enterotoxins kit, which can detect both Nhe and Hbl enterotoxins. Ceuppens et al., [111] compared the three detection kits using gastrointestinal samples to verify their suitability for *B. cereus* enterotoxin diagnosis. They highlighted that BCET-RPLA kit requires a dilution step in order to avoid false-positive outcome but present a low LOD. LOD of Hbl in gastrointestinal samples is similar to the limit described by the manufacturer in contrast with Nhe. Furthermore, they emphasized the inability of the kits to detect all the enterotoxin polymorphisms. Two out of these three kits are no more commercially available.

Other strategies were based on an indirect enzyme immunoassays (EIAs) using high-affinity antibodies. Dietrich et al., [112] compared EIA to detect Nhe in culture supernatants with a sandwich enzyme immunoassay. Moravek et al., [96] used the latter-mentioned technique in comparison with EIAs, cytotoxicity tests and PCR for the enterotoxin detection in *B. cereus* strains isolated from meat, milk, baby food and clinical environments. The results suggested that Nhe production was high in *B. cereus* strains causing food poisoning.

A chemical approach based on MALDI-TOF/MS was explored in order to detect the different diarrheal toxins produced by *B. cereus*. Tsilia et al., [113] applied this approach, and used characteristic *m/z* spectral positions to identify CytK1 and Nhe proteins in supernatants of different *B. cereus* cultures. Results were obtained with the BDE VIA kit. MALDI-TOF/MS showed a higher specificity, sensitivity and confidence in enterotoxin detection compared to the lateral flow kit. However, the MS method requires labor-intensive preparations, high investment and maintenance.

A different detection strategy was explored by Gabig-Ciminska et al., [107] who developed an electric chip for the screening of haemolysin producing *B. cereus*. The chip-recognized hblC and hblA genes. The first step of the test consisted of a sandwich formation between the DNA target, the capturing probes immobilized onto magnetic beads by amino-link, and the specific detection probes labeled with biotin. The formed sandwich was detected by a silicon chip-based potentiometric cell, using extravidin-alkaline phosphatase complex as an enzymatic label. The addition of the enzymatic substrate pAPP enabled for amperometric detection of the reaction product. The biosensor detected specific signals from 20 amol of bacterial DNA in less than 4 h. This low LOD allowed for *B. cereus* enterotoxin-encoding genes detection in crude cell extracts without preceding nucleic acid amplification [107].

**Figure 7:**
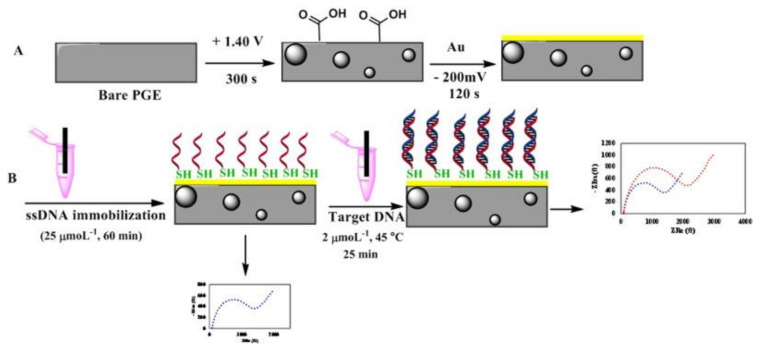
Schematic presentation of the immobilization of the ssDNA on a pencil graphite electrode modified with nano-gold (**A**) and its hybridization with *B. cereus* genomic DNA (**B**) (Reprinted with Permission from [60]).

Recently, other electrochemical biosensors were developed to detect diarrheal *B. cereus* strains. For instance, Izadi et al., [60] developed a gold nanoparticle modified pencil graphene electrode (AuNPs-PGE) for screening diarrheal *B. cereus* strains in milk and infant formula using electrochemical impedance spectroscopy (EIS). A ssDNA probe targeting *nheA* gene was selected as a recognition element and immobilized on the electrode surface. For this purpose, the probe was modified by a thiol group (HS-(CH_2_)_6_ at the 5′ end to enable its self-assembling onto the gold nanoparticles and electro-deposited over a pencil graphite electrode, as illustrated in Figure 7. Hybridization of the target DNA against the immobilized ss-DNA increased the charge transfer resistance of the biosensor enabling the detection of *B. cereus*. A LOD of 100 CFU/mL was achieved using genomic DNA extracted from bacteria from spiked infant formula.

### 4.3. Detection of Other B. cereus Pathogenic Factors

The detection of Nhe and Hbl enterotoxins provides an indication of the toxicity of a strain but are not sufficient to totally discriminate between pathogenic and non-pathogenic strain potentials [97]. Indeed, several studies have shown that the Nhe production by hazardous strains is variable and that non-pathogenic strains can also produce it in large quantities [90,114].

In addition to Nhe and Hbl toxins, *B. cereus* produces several compounds (degradation enzymes, cytotoxic factors, hemolysins, proteases, cell-surface proteins) that might also contribute to virulence [105,115]. For instance, InhA1 and NprA are secreted metalloproteases, which interfere with the host immune defenses and play a role in the virulence of *B. cereus* [116,117,118,119]. Other enterotoxins, such as enterotoxin T (BcET) and enterotoxin FM (EntFM) were reported to be involved in foodborne illness [120,121]. The pore-forming Hemolysin II (HlyII) toxin induces macrophage apoptosis and allows *B. cereus* to bypass the host immune defenses [121,122]. The importance of HlyII in the pathogenicity of *B. cereus* [122] has been demonstrated in several models, invertebrates and mammals [123].

Although the role of these proteins are not fully established, several authors applied molecular techniques to detect their genes together with the other toxin-encoded genes in FBO or clinical strains in a multiplex detection format [90,92,109,124]. For instance, [95] characterized *B. cereus* toxigenic strains by means of PCR technique in several food samples from local restaurants and supermarkets, using *cytK*, *bceT* and genes encoring Hbl and Nhe complexes as biomarkers. They showed that almost 70% of the isolates held the *bceT* gene. The high presence of the enterotoxin encoded by the *bceT* in the foodstuffs suggested its importance in *B. cereus* virulence. The search for the *hlyII* gene in a large *B. cereus* collection including FBO-related strains and human clinical samples, revealed that the *hlyII* gene is present in approximately a quarter of *B. cereus* strains [125,126] and those which carry this gene are described as pathogens [126].

A short list of the main strategies explored to detect *B. cereus* toxins is displayed in Table 3.

However, to the best of our knowledge, common markers to all pathogenic strains have not been described. For example, some genes considered as virulence factors such as *nhe*, *pi-plc* and *entFM* are present in all *B. cereus* strains, pathogenic or not [23,90,97,131,132], while well-known virulence factors such as *hlyII*, *cytk-1* and *ces* are not characteristic of all pathogenic strains. It is even apparent that many strains causing infections do not possess any of them [23,126].

Taken together, the virulence potential of *B. cereus* is likely multifactorial with a combination of known and maybe unknown factors been responsible for the various pathologies associated with *B. cereus* infections. This implies that only multiplex detection of *B. cereus* genes should be applied to determine the pathogenic potential of a particular strain. Currently, there are no advanced detection methods developed to detect these potential factors at the protein level. In addition, these factors are not yet searched in foodstuff or in clinical sample on a routine basis. Thereafter, innovative multiplexed technologies need to be developed for the detection of pathogenic toxins of *B. cereus*, in order to provide a diagnostic tool to improve both clinical diagnosis and food safety.

## 5. Conclusions

*Bacillus cereus* is an important cause of foodborne pathogenesis because it contaminates numerous foods including infant formula, infant rice cereal, milk and dried milk products, rice (including cooked rice), eggs, meat, and spices. Pathogenic *B. cereus* strains cause food poisoning or even severe human infectious diseases with an infective dose as low as 10^3^ CFU/g of food. Although *B. cereus* is currently searched only in infant formula, it is recommended as a suitable microbiological safety indicator for food products. The traditional detection methods that enumerate *B. cereus* colonies on selective agars are labor, cost-intensive and time-consuming (from 2 to 7 days for confirmation). Furthermore, they cannot detect injured or VBNC cells. Subsequently, they are not suitable for the needs of the food industry. Molecular methods, immunoassays and chemical methods have been developed to identify *B. cereus* strains that produce toxins such as cereulide, cytotoxin K, Hbl and Nhe. To reveal the prevalence and characteristics of B. cereus in various foods PCR-based methods targeting one or several toxin genes like *hblA*, *hblC*, *hblD*, *nheA*, *nheB*, *nheC*, *cytK*, *entFM*, *bceT*, *hlyII*, and *ces* are usually applied. In the past decade, electrochemical, optical and colorimetric biosensors exhibiting a rapid, low cost and high sensitivity have been developed for specific detection of *B. cereus* in food products. Considering that biosensors may exhibit high reproducibility and stability and can be miniaturized to a portable multiplex format, they can constitute a suitable tool to reduce risk of *B. cereus* contamination. Regular screening of foodstuffs for *B. cereus* will enhance the perception about contamination and provide a theoretical basis for developing effective measures to reduce product recalls and substantial economic loss. At the hospital level, an accurate diagnosis will help improving patient care and health.

Presented biosensors based on various recognition elements such as DNA probe, antibodies, aptamers, or phage possess advantages and disadvantages, often trading efficiency and robustness in exchange for specificity, affordability or limit of detection. For instance, antibodies are used in biosensors and assays for the detection of *B. cereus* cells or their toxins. Although it is usually easy to perform such tests because they may be performed without biomarker purification or special sample pretreatment, antibodies may show cross-reactivity, which potentially reduces their specificity. Peptides and aptamers represent attractive alternatives to expensive antibodies for a specific bacterial targeting since they offer not only good sensitivity and specificity but also high stability and chemical resistance. Especially aptamers selected by the cell-SELEX technology are very promising tools for bacterial cells recognition [133,134]. However, up to now, aptamer-based biosensors have been developed only for *B. cereus* spores detection [6]. Finally, DNA based biosensors make possible to differentiate specific *B. cereus* strains or to detect their toxin-producing genes. To enable efficient detection, DNA probes should be designed carefully to combine maximal specificity and minimal non-specific interaction, and to avoid any formation of the secondary structure of the probe, which will prevent its access to targets. Although biosensors offer great improvements compared to classical culture-based methods, there are many challenges to overcome, especially those involving their stability. We strongly believe that an interdisciplinary approach involving analytical chemistry, microbiology, materials science, biochemistry, and molecular biology will allow for the development of proposed systems at the proof-of-concept stage to reach the market in the future.

## Figures and Tables

**Table 1:** Detection methods for *B. cereus* vegetative cells.

Recognition Element	Biomarker	Method	Limit of Detection (LOD)	Matrice	Reference
Polyclonal antibodies	Whole-cell	Direct-charge transfer biosensor	10^1^ to 10^2^ CFU/mL	Pure culture	[70]
Polyclonal antibodies	Whole-cell	Direct-charge transfer biosensor	35–88 CFU/mL	Inoculated sprout, strawberries, lettuce, tomatoes, fried rice and corn.	[61]
DNA probe	DNA	PCR-TTGE; RAPD-PCR; rep-PCR		Food, patients and pesticides	[41]
DNA probe	*motB* gene	Electrochemical biosensor		Pure culture	[63]
					
Antibodies	Whole-cell	IMLN* Magnetic sensor	10 CFU/mL	Milk	[71]
					
Primer-probe	16S rRNA	RTi-PCR	16.5 CFU/mL	Pasteurized food	[39]
Primer	*gyrB* gene	PCR and RAPD PCR		Vended hot chocolate powder and hot-drinks vending machine	[38]
Polyclonal antibodies	Whole-cell	Cyclic voltammetry with IMS*	40 CFU/mL	Pure culture	[65]
Phage endolysin CBD	Whole-cell	Surface Plasmon Resonance (SPR)	10^2^ CFU/mL	Pure culture	[67]
Primer	Cereulide sinthetase gene (*cesB*)	PMA-asPCR-AuNPs colorimetric assay	9.2 × 10^1^ CFU/mL 3.4 × 10^2^ CFU/mL	Pure culture Milk	[36]
Antibody or Bacteriophage CBD	Wholecell	ATP bioluminescence assay	10^1^ CFU /mL	Pure culture	[68]
Probe	DNA	Electrochemical DNA biosensor	2.0 × 10^−15^ M	Pure culture	[46]
Molecularly Imprinted Polymer	Whole-cell	Quartz crystal microbalance	10^7^ CFU/mL	Water	[72]

* IMLN = Immunomagnetic beads-immunopolisomal nanovesicles; IMS = Immuno magnetic separation.

**Table 2:** Detection methods for cereulide and emetic *B. cereus* vegetative cells.

Recognition Element	Biomarker	Method	Matrice	Reference
Primer	*ces* gene	mPCR	Pure culture	[90]
Primer and probe	*ces* gene	RTi-PCR	Pure culture	[73]
Cereulide; valinomycin	*m/z* 1170.7 *m/z* 1128.5	LC-MS	Cooked rice; Chinese noodle dish	[84]
Primer	*ces* gene	mRTi-PCR	Spiked baby food	[91]
Cereulide; cereulide-C13	*m/z* 1170.7 *m/z* 1176.7	UPLC-ESI*-MS/MS	Rice; pasta	[79]
Primer	*ces* gene	PMA*-mPCR	Spiked baby cereal; pasteurized milk rice	[92]
Mass/Charge	Cereulide	LC-MS/MS	Spiked rice; cream pastry; mini pancakes; infant formula	[93]
Mass/Charge	Cereulide; cereulide variant	*MALDI-TOF/MS	Rice; milk; ready-to-eat meals	[87]
Mass/Charge	Cereulide	*MALDI-TOF/MS	Pure culture	[86]
Primer and hairpin probe	*ces* gene	Fluorescence assay combined with PCR, CHA* and GO*	Pure culture Milk	[88]

Note: * ESI= Electrospray; PMA = Propidium monoazide; MALDI-TOF = Matrix-assisted laser desorption/ionization-time of flight; CHA = catalytic hairpin assembly; GO = graphene oxide.

**Table 3:** Detection method for *B. cereus* enterotoxins and other pathogenic factors.

Recognition Element	Biomarker	Method	Matrice	Reference
Primer	*hblA,hblD,hblC/nheA,* *nheB,nheC/bceT*	PCR	Pure culture	[127]
Antibodies	*nheA,nheB,nheC*	EIAs	Infant formula and dried milk products	[112]
Primer and probe	*hblC*	Electrochemical biosensor	Pure culture	[128]
Antibodies, primer	HBL-L2/*nheA,* *nheB,nheC*	EIAs, sandwich enzyme immunoassay, cytotoxicity test, PCR	Remnant connected to food-borne outbreak	[96]
Primer and probe	*hblA,hblD,hblC/nheA,* *nheB,nheC/cytK/ces*	Electric DNA array	Pure culture	[129]
DNA probe	*Pc-plc* gene	RTi-PCR	Artificially contaminated liquid eggs and infant formula	[114]
Antibodies, primer	*hblA,hblD,hblC/nheA,* *nheB,nheC/cytK1/ces*	Immunoassay, mPCR, cytotoxicity test	Pure culture	[90]
Monoclonal antibodies	HBL-L2/nheB	Duopath^®^ kit	Artificially contaminated baby food, rice	[130]
Primer	*hblA,hblD,hblC/nheA,* *nheB,nheC/bceT/ cytK1/ces*	PCR, BCET-RPLA	Rice, yogurt, pasta, cake	[95]
Antibodies	NheA, NheB/Hbl-L2	BDE VIA^TM^; BCET-RPLA; Duopath^®^ kit	Lasagna, human faeces, potatoes	[111]
Mass/charge	Cytk1 and NheA	MALDI-TOF/MS	Pure culture	[113]
Primer	*hblD/nheA/entFM/cytK/ces*	PMA-mPCR	Pure culture	[92]
Probe	*nheA*	Electrochemical DNA-based biosensor	Milk and infant formula	[60]
Antibodies, primer	*nheA/hblC/entFM/cytK*	TECRA, mPCR	Pasteurized milk	[108]
